# Parity and serum lipid levels: a cross-sectional study in chinese female adults

**DOI:** 10.1038/srep33831

**Published:** 2016-09-20

**Authors:** Haichen Lv, Xiaolei Yang, Yong Zhou, Jing Wu, Henghui Liu, Youxin Wang, Yuanming Pan, Yunlong Xia

**Affiliations:** 1 Department of Cardiology, First Affiliated Hospital of Dalian Medical University, Dalian, China; 2 Department of Neurology, Beijing Tiantan Hospital, Capital Medical University, Beijing, China; 3 School of Public Health, Dalian Medical University, Dalian, China; 4 Beijing Recdata Technology Co. Ltd., Beijing, China; 5 Beijing Municipal Key Laboratory of Clinical Epidemiology, School of Public Health, Capital Medical University, Beijing, China

## Abstract

Reproductive factors have been shown to correlate with lipid metabolism. The aim of this study was to investigate the relationship between parity and serum lipid levels in community-based Chinese female adults. A total of 4,217 female participants were enrolled. Parity was recorded according to questionnaire and serum lipid profile, including triglycerides (TG), total cholesterol (TC), low density lipoprotein cholesterol (LDL-C), and high density lipoprotein cholesterol (HDL-C), was measured. Logistic regression models were used to analyze the association of parity to serum lipid levels, while adjusting for demographics and metabolic risk factors. Parity in this population ranged from 0 to 7. After adjusting for potential confounders, it indicated that females with more than 2 parities appeared to be less likely to suffer from abnormal serum TC level compared with nulliparae (parity = 2, odds ratio (OR) = 0.457, 95% confidence interval (CI) = 0.284–0.736; parity ≥ 3, OR = 0.363, 95% CI = 0.202–0.653). These findings suggested that parity could correlate with lipid metabolism in Chinese women. Individuals with higher parity appeared to have a lower total cholesterol in blood.

Dyslipidemia, which represents a series of lipid metabolism disorder, plays a role in the development of atherosclerotic cardiovascular diseases (ASCVD)^1^,^2^. Except for dyslipidemia and some established risk factors of ASCVD^2^, several studies have hypothesized that reproductive factors of female can also be related to ASCVD^3^,^4^,^5^,^6^,^7^,^8^,^9^,^10^,^11^,^12^,^13^.

Pregnancy and delivery are important events in the life of a woman. Gestation leads to a cascade of physiological change in sex hormones levels, hemodynamics, oxidative stress, and so on, which can exert complex influences on major organ systems^5^, therefore, may also have long-term implications for women’s health^7^,^12^. Prior researches have suggested that reproductive factors might have an additive effect with later glycolipid metabolism^10^,^11^,^12^,^14^,^15^,^16^,^17^,^18^,^19^, but their inner-link is still inconsistent. Unfortunately, only limited studies^8^,^17^,^18^,^20^,^21^ devoted substantial progress to evaluating the relationship between childbearing history and the occurrence of female dyslipidemia.

Herein, we performed this study to explore the association between parity, as reflected by number of birth, and serum levels of lipids, including triglycerides (TG), total cholesterol (TC), low-density lipoprotein cholesterol (LDL-C), and high-density lipoprotein cholesterol (HDL-C), in Chinese female adults.

## Results

### Baseline characteristics

As displayed in Table 1, of the studied 4,217 female subjects, parity ranged from zero to a maximum of seven. Nulliparae (N = 543) constituted 12.88% of this cohort, while 99.88% subjects (N = 4,212) in our study had ≤4 children. Clinical characteristics of all participants were illustrated according to parity in Table 2. Compared with nulliparae, multiparae were slightly older as parity increased. Lower prevalence of hypertension, diabetes, dyslipidemia, myocardial infarction (MI), stroke, along with lower body mass index (BMI) and higher estimated glomerular filtration rate (eGFR) was demonstrated in the groups of lower parities. Women with more children used more commonly antihypertensive, antidiabetic, and antihyperlipidemic drugs but consumed less alcohol. Those with two children had higher tobacco use. There was no statistical difference in estrogen replacement therapy between groups. Moreover, the groups of lower parities had higher proportions of subjects with advanced education degrees and higher income.

### Parity and serum lipid levels

As shown in Table 3, without adjustment for potential confounders, all the serum levels of TG, TC, LDL-C, and HDL-C varied with statistical significance in every category. Serum levels of TG, TC, and LDL-C increased with parity rising up; on the contrary, HDL-C level was lower in women with more children.

### The correlation between parity and disordered serum lipid levels

Although the differences were observed in serum levels of TG, TC, LDL-C, and HDL-C between groups (Table 3), statistical correlation only could be found between parity and abnormal TC level while adjusting by logistic regression analysis (Table 4). More than two parities appeared to be a protective factor of abnormal serum level of TC. Since parity and serum lipid levels might interact with different confounders, four consecutive models of multivariate adjustment were created. The first model adjusted for age, the second included lifestyle and chronic disease variables (BMI, smoking, diabetes, hypertension, eGFR). The third model was obtained with socioeconomic factors such as education level and income. Moreover, we performed the fourth model wherein cardiovascular diseases and medication history (MI, stroke, alcohol use, antihypertensive medication, insulin or oral hypoglycemic agent, lipid-lower therapy, and estrogen replacement) were taken into consideration. As shown in Table 4, there was a significant protective effect against abnormal TC level in the subjects with two (odds ratio(OR) = 0.457, 95% confidence interval(CI) = 0.284–0.736) or more children (OR = 0.363, 95% CI = 0.202–0.653) after full adjustment.

## Discussion

To our knowledge, this is the first observational study to explore the potential relationship between parity and serum lipid levels among general Chinese adult female population specially. After adjusting for potential risk factors (including age, BMI, smoking, diabetes, hypertension, eGFR, education, income, MI, stroke, use of alcohol, antihypertensive medication, insulin or oral hypoglycemic agents, lipid-lower therapy, and estrogen replacement), there was a trend that multiparae with more than two children had a lower risk of hypercholesterolemia. Contrast to previous studies^6^,^17^,^18^,^22^, we demonstrated that parity acted on the level of total cholesterol entirely, but not influenced lipid metabolism by TG, LDL-C, or HDL-C separately.

Lipids, such as cholesterol and triglycerides, are carried in lipoproteins which transport lipids to tissues for energy utilization, lipid deposition, steroid hormone production and bile acid formation. Abnormal lipid metabolism, as well as dyslipidemia, is a significant precipitating factor of atherosclerosis and ASCVD^1^,^2^,^23^. Higher serum levels of TC, TG, or LDL-C and lower level of HDL-C promote atherosclerosis plaque formation.

The potential relationship between pregnancy and lipid levels has been stated in two studies early in 1980s^20^,^21^. During pregnancy, serum TG, TC, and LDL-C increase progressively. However, during postpartum period, unlike serum TG decreasing rapidly, the elevations of TC and LDL-C can last longer before dropping back to baseline^22^. Pregnancy has also been confirmed to have an adverse influence on HDL-C, which peaks at mid-gestation but falls to a level below baseline after that^22^,^24^, furthermore, the decrease of HDL-C tends to continue after delivery^12^.

Ever since, few studies^6^,^17^,^18^,^22^ have focused on the relationship between parity and lipid levels in western countries, but the aforementioned association is still inconsistent. In a cohort of 516 female adults^17^, Deslypere *et al.* first reported that TC and LDL-C levels were lower but HDL-C/TC ratios were higher in women after first pregnancy than those after five or more pregnancies. Kritz-Silverstein *et al.*^18^ observed 1,275 subjects and found that parity was unrelated to lipid levels in those with fewer births, but women with five or more pregnancies had lower HDL-C levels. A research from Netherland^22^ suggested that there was a trend toward lower HDL-C levels and higher TC/HDL-C ratios with parity increasing in women aged ≥55 years. However, Wolff *et al.*^6^ found that women with one or two children had lower LDL-C levels, whereas their HDL-C levels were higher marginally.

Different from the results of formal studies^6^,^17^,^18^,^22^, findings from current study show a significant protective effect on the incidence of high TC disorder while parity reaches to two or higher. Our results may suggest that parity impacts on lipid metabolism in the life of a woman. Several potential mechanisms might have been proposed but the exact biologic mechanisms are not fully understood. At first, previous studies^5^,^6^,^7^,^8^,^22^,^25^ have confirmed the correlation between parity and ASCVD. Since cholesterol is considered an important risk factor in the development of ASCVD, it is possible that parity can be associated with ASCVD via hypercholesterolemia. Secondly, relative insulin resistance develops in normal pregnancy and assists supplying energy to the baby^8^,^11^,^26^,^27^,^28^, which leads to higher levels of free fatty-acids in blood. These may result in complicated adverse metabolic changes, such as abnormal glucose tolerance, dyslipidemia, and obesity^25^,^26^,^29^. After delivery, most of the metabolic patterns return to baseline by a protective compensation reaction, which might be enhanced and last for long term by repeated pregnancies. Thirdly, it is proposed that each pregnancy “resets” ovarian function permanently^30^. Several studies^14^,^15^,^31^ have demonstrated that estrogen and its analogues refer to lipid metabolism by interfering with lipid oxidation and secretion. Hence, exposure to estrogen may also contribute to labile lipid levels. From another perspective, cholesterol is necessary for steroidogenesis, so repeated pregnancies may expend more cholesterol to produce hormones. Moreover, pregnancy complications, such as gestational diabetes, pregnancy hypertension, preeclampsia, and preterm birth, may lead to lower parity as women once with these complications may be less likely to have more children. However, these women are more likely going to have metabolic disorders such as hypertension, diabetes, as well as atherogenic lipid profiles^32^,^33^,^34^. Furthermore, socioeconomic and lifestyle factors cannot be ignored. Mothers with higher parities are more often in lower social classes with lower incomes, lower education levels, larger families^8^, and younger age at first pregnancy^7^. These women are more likely to be occupied in physical jobs and be on lower-nutrition diets in China, which can also cause additional effects on serum cholesterol level.

Apart from the probable biological mechanisms mentioned above, another factor which may cause our findings in Chinese women different from those observed in Western country is the grouping strategy of parity number. As a result of birth control policy in China, few women enrolled bear more than four children, so we classified parity into four categories as zero, one, two, and three or more in order to balance the sample size of each group. In contrast, different findings in western studies may be attributable to more haphazard parity and grouping strategy^6^,^17^,^18^,^22^. In fact, a recent study^5^ has identified that a J-shaped association could be observed in the non-linear dose-response meta-analysis of parity number and cardiovascular diseases (CVD) mortality, wherein the relationship between parity number and CVD mortality appeared to follow an inversely linear dose-response pattern until parity number reached four live births. Since parity less than or equal to four accounted for 99.88% participants in the present study, our findings also support this prior work since hypercholesterolemia can be treated as a risk predictor of CVD prognosis.

Our research has several strengths. This study aimed to address one aspect of the relationship between reproductive factors and lipid metabolism, which had been relatively under-investigated in Asian women, especially in Chinese population. The study included a total of 4,217 participants, ensuring sufficient statistical power to detect and verify the association between parity and serum lipid levels. It was also less biased as a community-based design was used. Although evidence from long-term randomized trials is more ideal, these studies are too difficult to be conducted into practice, especially considering reproductive factors.

This study is cross-sectional and might be affected by reverse causality and survivor bias. Therefore, several limitations should be acknowledged. Firstly, the current study is based on a population consist in of the participants of Population based Cohort Study in Outcome of Phased Progression of Atherosclerosis (PERSUADE), most of the participants were employees, retirees and relatives of Jidong coal mine industry; thus, the results cannot be generalized to all Asian women. Secondly, we didn’t take all the probable related variables, such as menopause, delivery mode, and so on, into consideration, which might become potential confounders. Thirdly, the possibility of residual confounders remains. As mentioned above, parity might associate with potential pregnancy complications and lifestyle factors, the observed correlation between parity and lipid metabolism is unlikely to consider these aspects impeccably.

In conclusion, after adjusting for potential related factors, this study shows that parity is associated with lipid metabolism in Chinese female adults. Individual with moderate higher parity tends to have a decreased risk of abnormal total cholesterol level. However, the nature of such a relationship requires more evidence and further investigation.

## Methods

### Study design and population

The study is based on a population consisting of the participants of Population based Cohort Study in Outcome of Phased Progression of Atherosclerosis (PERSUADE). From July 2013 to August 2014, all residents aged 18 years and above from Jidong community were invited to participate in this study. The community is geographically located in Tangshan, Hebei, China, and is mainly comprised of employees of the Jidong Oilfield Inc. and their family members. Of the all 10,043 adult residents in Jidong community, 9,078 participated in this study and provided informed consent, including 4,310 females. A total of 4,217 individuals remained in the statistical analysis after excluding 93 subjects with different missing data.

### Assessment of serum lipid levels

Lipid levels of the subjects enrolled were measured along with the other indicators at the same time at baseline. Venous blood samples were obtained via venipuncture of the large antecubital veins after overnight fasting 10–12 hours. The levels of serum lipids, including TG, TC, LDL-C, and HDL-C, were determined with auto-analyzer (AU400; Olympus, Tokyo, Japan) according to manufacturer’s instruction at the central laboratory of Jidong Oilfield Hospital. In this study, we defined TC disorder as TC ≥ 5.18 mmol/L, TG disorder as TG ≥ 1.7 mmol/L, LDL-C disorder as LDL-C ≥ 3.37 mmol/L, and HDL-C disorder as HDL-C < 1.04 mmol/L.

### Assessment of demographic variables and other indicators

A standardized and structured questionnaire to gather information on subjects’ demographic characteristics, metabolic risk factors, and medical conditions were administered by well-trained interviewers. In the questionnaire, parity was classified into four categories as zero, one, two, and three or more. Physical examinations were performed by physicians. According to self-reported information, smoking status was classified as “current smoker or quitting less than one year”, or “non-smoker or quitting more than one year”, and alcohol use was defined as daily intake of at least 100 ml of liquor (equivalent to 240 ml of wine or 720 ml of beer) for more than one year. The information of disease history included hypertension, diabetes, dyslipidemia, myocardial infarction(MI), stroke, and so on. Hypertension was defined as presence of a history of hypertension, or using antihypertensive medication, or a systolic pressure ≥140 mmHg, or a diastolic blood pressure ≥90 mmHg. Diabetes was defined as a self-reported history, currently treated with insulin or oral hypoglycemic agent, or fasting blood glucose level ≥7.0 mmol/L. Dyslipidemia was defined as a self-reported history, serum levels of TC ≥ 5.18 mmol/L or TG ≥ 1.7 mmol/L or LDL-C ≥ 3.37 mmol/L or HDL-C < 1.04 mmol/L or current use of lipid-lower therapy according to Chinese guideline. History of pregnancy complications (e.g., gestational diabetes, pregnancy hypertension, preeclampsia, and preterm birth) was classified as “yes” or “no” altogether. Medication histories, including the use of antihypertensive medication, insulin or oral hypoglycemic agent, lipid-lower therapy, and estrogen replacement were distinguished as “yes” or “no” based on self-reported information. BMI were defined based on measured heights (accurate to 0.1 cm) and weights (accurate to 0.1 kg), and calculated as the body weight (kg) divided by the square of height (m^2^). Estimated glomerular filtration rate (eGFR) was calculated with an equation adapted from the Modification of Diet in Renal Disease (MDRD) equation^35^. Education level was categorized as “illiteracy, primary or middle school”, “high school”, and “college, university or above”. Average monthly income of each family member was categorized as “≤¥1,000”, “¥1,001–3,000” and “>¥3000”.

### Statistical analyses

Variables collected at baseline were analyzed. Statistical analyses were performed using SPSS software, version 20.0 (SPSS Inc.,Chicago, Illinois). Continuous variables were described by mean ± standard deviation (SD) and were compared using ANOVA analysis. Categorical variables were described by percentages and were compared using Chi-square tests. Logistic regression analysis was used to estimate odds ratios (OR) and 95% confidence intervals (CI) in order to assess the association between parity and lipid levels with other parameters such as age, hypertension, diabetes, and BMI adjusted. Since there was no statistical difference of pregnancy complications (e.g., gestational diabetes, pregnancy hypertension, preeclampsia, and preterm birth) among each category of parity and further adjustment for this variable did not alter our findings (<10% change in OR), therefore, pregnancy complications were not included in the final models in order to maintain model stability. All statistical tests were 2-sided, and *P* < 0.05 was accepted as statistically significant.

### Ethical approval

Our study was conducted according to the guidelines of Helsinki Declaration. Ethical approval for the research protocol and written informed consent were approved by the Ethics Committee of Jidong Oilfield Inc Medical Centers prior to the study initiation. Written informed consent was obtained from all participants. All the experiments described here were performed in accordance with the approved guidelines.

## Additional Information

**How to cite this article**: Lv, H. *et al.* Parity and serum lipid levels: a cross-sectional study in chinese female adults. *Sci. Rep.* **6**, 33831; doi: 10.1038/srep33831 (2016).

## Figures and Tables

**Table 1:** Parity distribution in this study.

Parity	Frequency	Percentage	Cumulative Frequency	Cumulative percentage
0	543	12.88	543	12.88
1	2983	70.74	3526	83.61
2	544	12.90	4070	96.51
3	121	2.87	4191	99.38
4	21	0.50	4212	99.88
5	2	0.05	4214	99.93
6	1	0.02	4215	99.95
7	2	0.05	4217	100.00

**Table 2:** Baseline characteristics of participants according to parity.

	Parity					P-value
	Overall	0	1	2	≥3	
Number, N (%)	4217 (100)	543 (12.88)	2983 (70.74)	544 (12.90)	147 (3.48)	
Age, mean ± SD, years	42.64 ± 12.89	28.01 ± 5.95	41.48 ± 10.32	57.84 ± 8.98	63.93 ± 7.11	<0.001
BMI, mean ± SD, kg/m^2^	23.46 ± 3.66	21.84 ± 3.65	23.34 ± 3.55	25.07 ± 3.43	25.60 ± 3.31	<0.001
Smoking, N (%)	64 (1.52)	5 (0.92)	41 (1.37)	16 (2.94)	2 (1.36)	0.028
Hypertension, N (%)	919 (21.79)	26 (4.79)	553 (18.54)	257 (47.24)	83 (56.46)	<0.001
Diabetes, N (%)	214 (5.07)	5 (0.92)	118 (3.96)	71 (13.05)	20 (13.61)	<0.001
eGFR, mean ± SD, ml/min/1.73 m^2^	96.47 ± 15.84	107.43 ± 12.98	97.34 ± 14.85	85.36 ± 13.68	79.45 ± 14.21	<0.001
MI, N (%)	12 (0.28)	0 (0.00)	3 (0.10)	6 (1.10)	3 (2.04)	<0.001
Stroke, N (%)	45 (1.07)	0 (0.00)	20 (0.67)	20 (3.68)	5 (3.4)	<0.001
Dyslipidemia, N (%)	1710 (40.55)	144 (26.52)	1139 (38.18)	331 (60.85)	96 (65.31)	<0.001
Alcohol use, N (%)	209 (4.96)	31 (5.71)	158 (5.30)	19 (3.49)	1 (0.68)	0.023
Antihypertensive medication, N (%)	357 (8.47)	1 (0.18)	178 (5.97)	139 (25.55)	39 (26.53)	<0.001
Insulin or oral hypoglycemic agent, N (%)	116 (2.75)	1 (0.18)	58 (1.94)	47 (8.64)	10 (6.80)	<0.001
Antilipemic agent, N (%)	47 (1.11)	0 (0.00)	27 (0.91)	11 (2.02)	9 (6.12)	<0.001
Estrogen replacement, N (%)	13 (0.31)	1 (0.18)	8 (0.27)	3 (0.55)	1 (0.68)	0.371
Education level, N (%)						
Illiteracy/primary/middle school	814 (19.30)	16 (2.95)	396 (13.28)	298 (54.78)	104 (70.75)	<0.001
High school	1035 (24.54)	66 (12.15)	763 (25.58)	176 (32.35)	30 (20.41)	
College/university/above	2368 (56.15)	461 (84.90)	1824 (61.15)	70 (12.87)	13 (8.84)	
Income, ¥/month^†^, N (%)						
<¥3000	1763 (41.81)	167 (30.76)	1099 (36.84)	391 (71.88)	106 (72.11)	<0.001
¥3000 to ¥5000	2136 (50.65)	324 (59.67)	1646 (55.18)	129 (23.71)	37 (25.17)	
>¥5000	257 (6.09)	34 (6.26)	204 (6.84)	18 (3.31)	1 (0.68)	

SD, standard deviation; BMI, body mass index; eGFR, estimated glomerular filtration rate; MI, myocardial infarction.

^†^Average monthly income of the family member.

**Table 3:** Analysis of serum lipid levels in different parity groups.

	Parity					P-value
	Overall	0	1	2	≥3	
TG ±SD, mmol/L	1.31 ± 0.98	1.07 ± 0.70	1.27 ± 0.98	1.62 ± 1.11	1.70 ± 1.01	<0.001
TC ± SD, mmol/L	4.42 ± 0.91	4.09 ± 0.77	4.38 ± 0.90	4.86 ± 0.89	4.91 ± 0.85	<0.001
LDL-C, mmol/L	2.41 ± 0.63	2.16 ± 0.53	2.38 ± 0.62	2.71 ± 0.63	2.77 ± 0.60	<0.001
HDL-C, mmol/L	1.29 ± 0.27	1.32 ± 0.27	1.29 ± 0.27	1.28 ± 0.27	1.24 ± 0.23	0.007

TG, triglycerides; SD, standard deviation; TC, total cholesterol; LDL-C, low density lipoprotein cholesteriol; HDL-C, high density lipoprotein cholesterol.

**Table 4:** Association between parity and serum lipid levels.

Type of dyslipidemia	Parity			
	0	1	2	≥3
TG disorder, N (%)	67 (12.34)	551 (18.47)	185 (34.01)	56 (38.10)
Model 1	1.00	0.797 (0.590–1.077)	0.853 (0.573–1.268)	0.770 (0.462–1.285)
Model 2	1.00	0.883 (0.634–1.230)	0.858 (0.558–1.318)	0.822 (0.475–1.421)
Model 3	1.00	0.876 (0.628–1.224)	0.823 (0.534–1.268)	0.799 (0.460–1.391)
Model 4	1.00	0.871 (0.622–1.220)	0.837 (0.540–1.296)	0.846 (0.484–1.479)
TC disorder, N (%)	42 (7.73)	514 (17.23)	183 (33.64)	54 (36.73)
Model 1	1.00	0.713 (0.493–1.030)	**0.502 (0.318**–**0.793)**	**0.361 (0.205**–**0.636)**
Model 2	1.00	0.698 (0.477–1.022)	**0.458 (0.285**–**0.735)**	**0.348 (0.195**–**0.623)**
Model 3	1.00	**0.679 (0.463**–**0.996)**	**0.454 (0.283**–**0.730)**	**0.361 (0.201**–**0.648)**
Model 4	1.00	0.682 (0.464–1.001)	**0.457 (0.284**–**0.736)**	**0.363 (0.202**–**0.653)**
LDL–C disorder, N (%)	11 (2.03)	207 (6.94)	77 (14.15)	24 (16.33)
Model 1	1.00	0.941 (0.486–1.820)	0.583 (0.274–1.241)	0.437 (0.183–1.043)
Model 2	1.00	0.884 (0.456–1.713)	0.529 (0.247–1.132)	0.430 (0.179–1.030)
Model 3	1.00	0.876 (0.452–1.700)	0.520 (0.243–1.113)	0.440 (0.183–1.057)
Model 4	1.00	0.857 (0.441–1.667)	0.509 (0.237–1.093)	0.433 (0.180–1.045)
HDL–C disorder, N (%)	83 (15.29)	492 (16.49)	97 (17.83)	23 (15.65)
Model 1	1.00	1.153 (0.874–1.521)	1.350 (0.897–2.032)	1.182 (0.656–2.128)
Model 2	1.00	1.247 (0.927–1.679)	1.370 (0.893–2.103)	1.197 (0.649–2.209)
Model 3	1.00	1.254 (0.928–1.694)	1.347 (0.875–2.075)	1.161 (0.622–2.166)
Model 4	1.00	1.256 (0.928–1.701)	1.345 (0.870–2.080)	1.209 (0.644–2.267)

Values are expressed as odds ratio (OR) and 95% confidence intervals (CI).

TG, triglycerides; TC, total cholesterol; LDL-C, low density lipoprotein cholesterol; HDL-C, high density lipoprotein cholesterol.

TG disorder, TG ≥ 1.7 mmol/L; TC disorder, TC ≥ 5.18 mmol/L; LDL-C disorder, LDL-C ≥ 3.37 mmol/L; HDL-C disorder, HDL-C < 1.04 mmol/L.

Model 1: adjusted for age.

Model 2: adjusted for age, BMI, smoking, diabetes, hypertension, and eGFR.

Model 3: adjusted for age, BMI, smoking, diabetes, hypertension, eGFR, education level, and income.

Model 4: adjusted for age, BMI, smoking, diabetes, hypertension, eGFR, education level, income, MI, stroke, use of alcohol, antihypertensive medication, insulin or oral hypoglycemic agent, lipid-lower therapy, and estrogen replacement.
